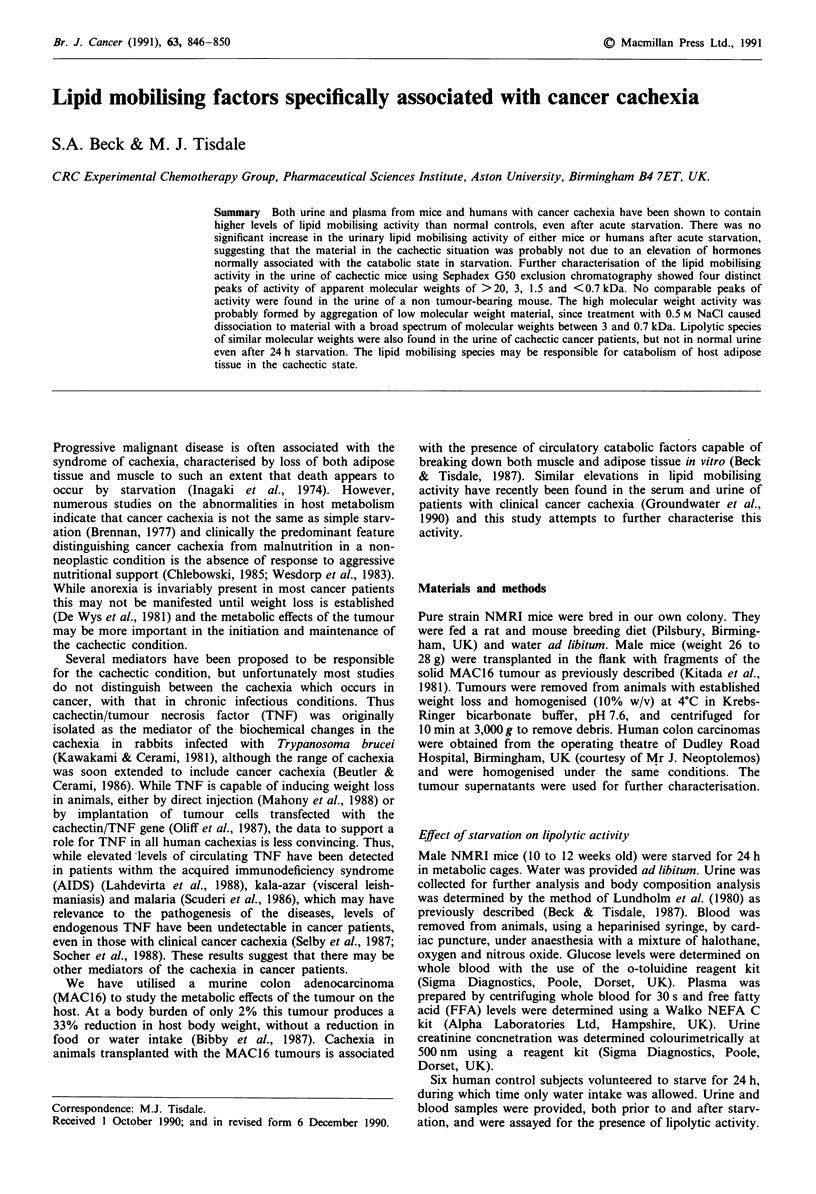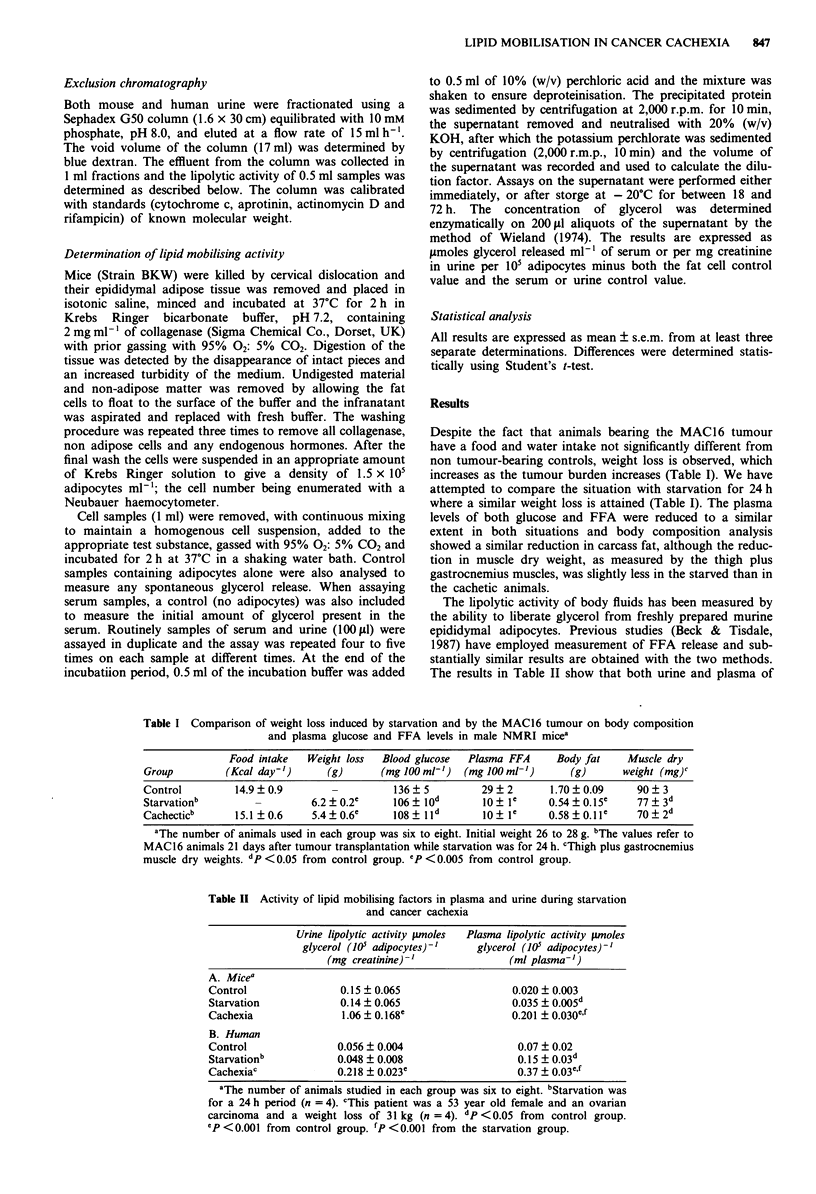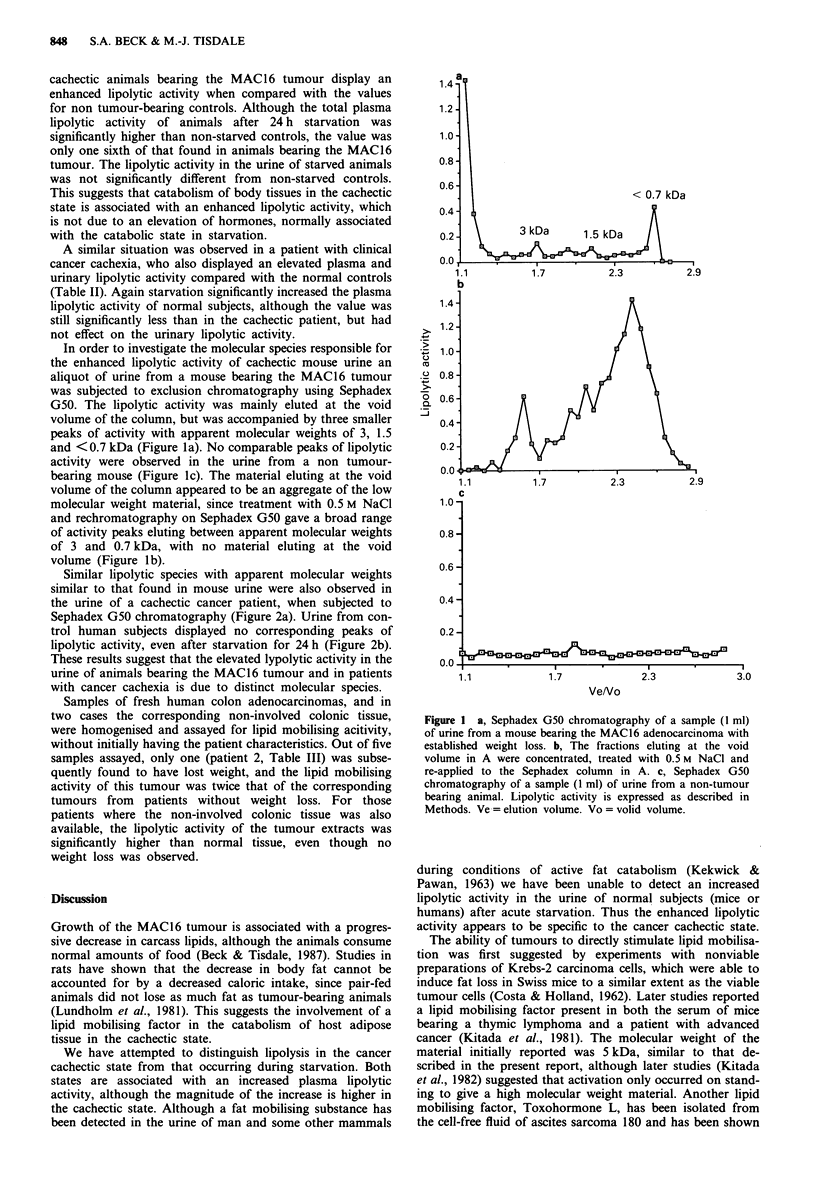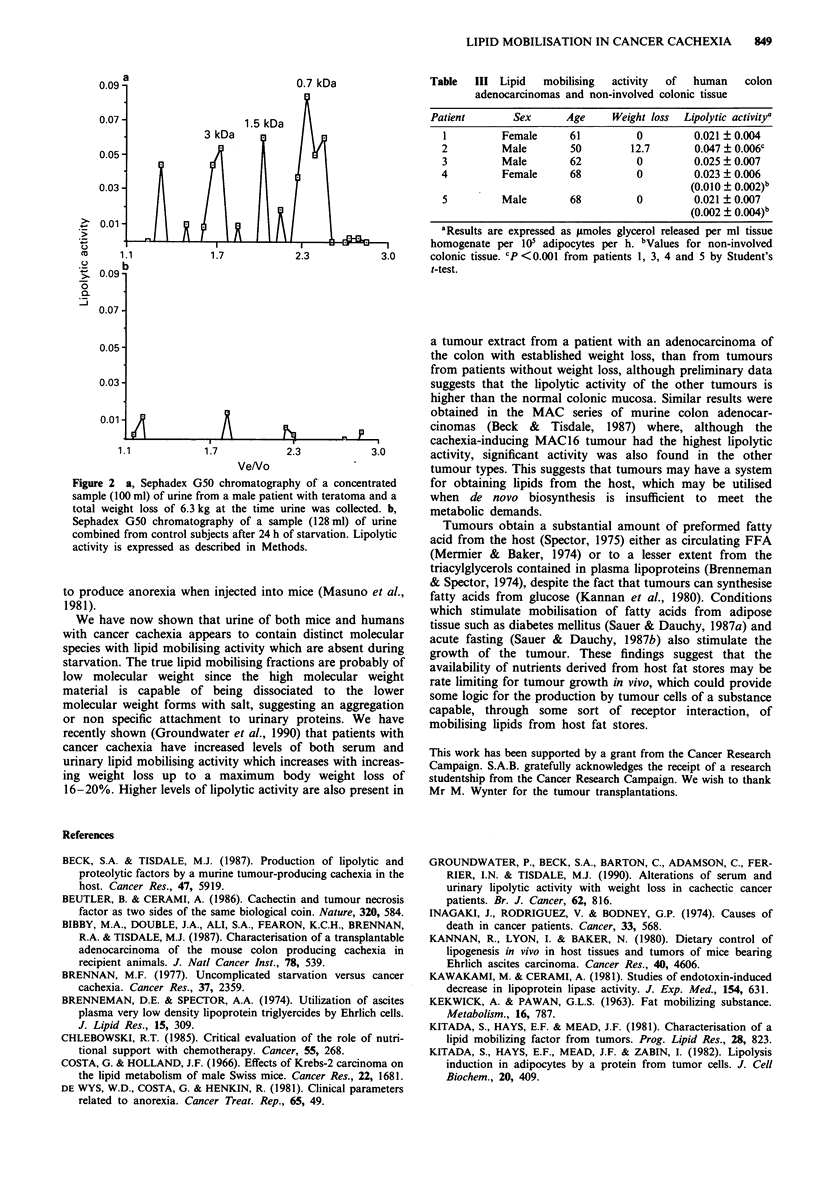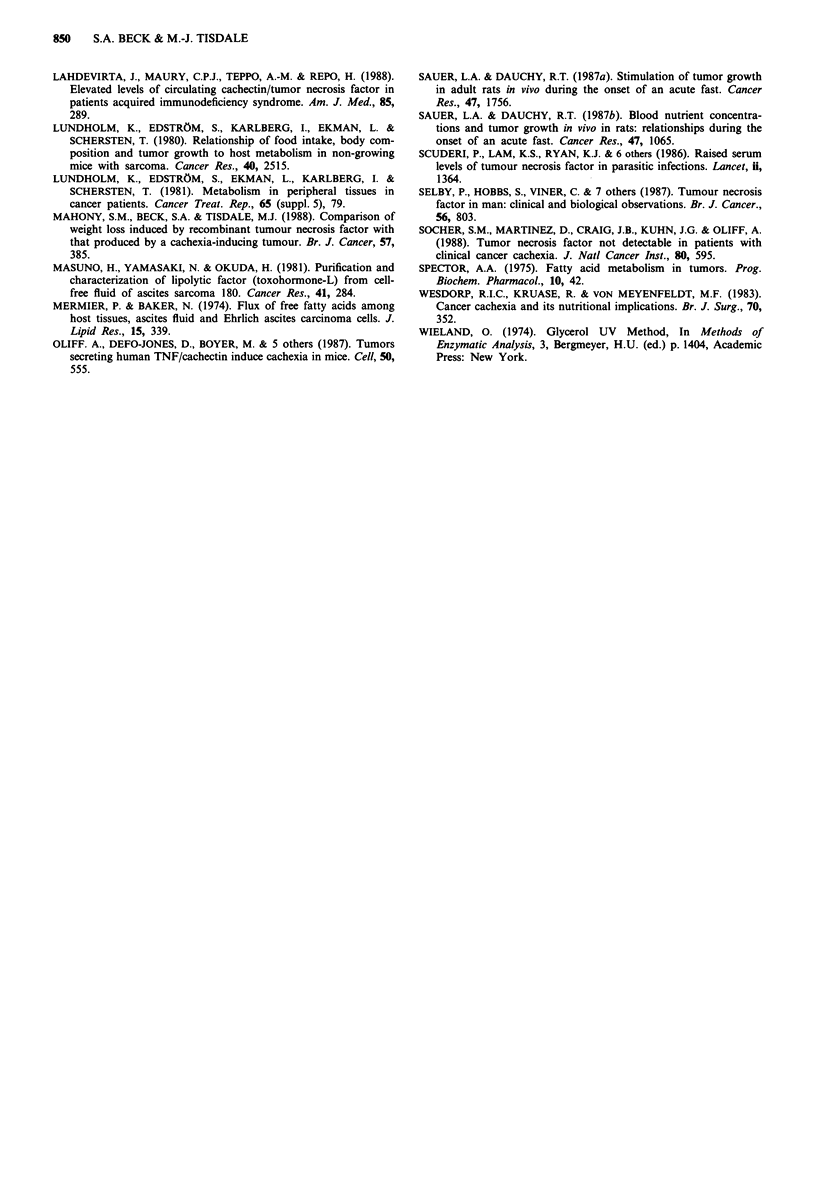# Lipid mobilising factors specifically associated with cancer cachexia.

**DOI:** 10.1038/bjc.1991.188

**Published:** 1991-06

**Authors:** S. A. Beck, M. J. Tisdale

**Affiliations:** CRC Experimental Chemotherapy Group, Aston University, Birmingham, UK.

## Abstract

Both urine and plasma from mice and humans with cancer cachexia have been shown to contain higher levels of lipid mobilising activity than normal controls, even after acute starvation. There was no significant increase in the urinary lipid mobilising activity of either mice or humans after acute starvation, suggesting that the material in the cachectic situation was probably not due to an elevation of hormones normally associated with the catabolic state in starvation. Further characterisation of the lipid mobilising activity in the urine of cachectic mice using Sephadex G50 exclusion chromatography showed four distinct peaks of activity of apparent molecular weights of greater than 20, 3, 1.5 and less than 0.7 kDa. No comparable peaks of activity were found in the urine of a non tumour-bearing mouse. The high molecular weight activity was probably formed by aggregation of low molecular weight material, since treatment with 0.5 M NaCl caused dissociation to material with a broad spectrum of molecular weights between 3 and 0.7 kDa. Lipolytic species of similar molecular weights were also found in the urine of cachectic cancer patients, but not in normal urine even after 24 h starvation. The lipid mobilising species may be responsible for catabolism of host adipose tissue in the cachectic state.


Br. J. Cancer (1991), 63, 846–850 © Macmillan Press Ltd., 1991

### Lipid mobilising factors specifically associated with cancer cachexia

S.A. Beck & M. J. Tisdale

CRC Experimental Chemotherapy Group, Pharmaceutical Sciences Institute, Aston University, Birmingham B4 7ET, UK.

**Summary** Both urine and plasma from mice and humans with cancer cachexia have been shown to contain higher levels of lipid mobilising activity than normal controls, even after acute starvation. There was no significant increase in the urinary lipid mobilising activity of either mice or humans after acute starvation, suggesting that the material in the cachectic situation was probably not due to an elevation of hormones normally associated with the catabolic state in starvation. Further characterisation of the lipid mobilising activity in the urine of cachectic mice using Sephadex G50 exclusion chromatography showed four distinct peaks of activity of apparent molecular weights of >20, 3, 1.5 and <0.7 kDa. No comparable peaks of activity were found in the urine of a non tumour-bearing mouse. The high molecular weight activity was probably formed by aggregation of low molecular weight material, since treatment with 0.5 M NaCl caused dissociation to material with a broad spectrum of molecular weights between 3 and 0.7 kDa. Lipolytic species of similar molecular weights were also found in the urine of cachectic cancer patients, but not in normal urine even after 24 h starvation. The lipid mobilising species may be responsible for catabolism of host adipose tissue in the cachectic state.

Progressive malignant disease is often associated with the syndrome of cachexia, characterised by loss of both adipose tissue and muscle to such an extent that death appears to occur by starvation (Inagaki *et al.*, 1974). However, numerous studies on the abnormalities in host metabolism indicate that cancer cachexia is not the same as simple starvation (Brennan, 1977) and clinically the predominant feature distinguishing cancer cachexia from malnutrition in a non-neoplastic condition is the absence of response to aggressive nutritional support (Chlebowski, 1985; Wesdorp *et al.*, 1983). While anorexia is invariably present in most cancer patients this may not be manifested until weight loss is established (De Wys *et al.*, 1981) and the metabolic effects of the tumour may be more important in the initiation and maintenance of the cachectic condition.

Several mediators have been proposed to be responsible for the cachectic condition, but unfortunately most studies do not distinguish between the cachexia which occurs in cancer, with that in chronic infectious conditions. Thus cachectin/tumour necrosis factor (TNF) was originally isolated as the mediator of the biochemical changes in the cachexia in rabbits infected with *Trypanosoma brucei* (Kawakami & Cerami, 1981), although the range of cachexia was soon extended to include cancer cachexia (Beutler & Cerami, 1986). While TNF is capable of inducing weight loss in animals, either by direct injection (Mahony *et al.*, 1988) or by implantation of tumour cells transfected with the cachectin/TNF gene (Oliff *et al.*, 1987), the data to support a role for TNF in all human cachexias is less convincing. Thus, while elevated levels of circulating TNF have been detected in patients withm the acquired immunodeficiency syndrome (AIDS) (Lahdevirta *et al.*, 1988), kala-azar (visceral leishmaniasis) and malaria (Scuderi *et al.*, 1986), which may have relevance to the pathogenesis of the diseases, levels of endogenous TNF have been undetectable in cancer patients, even in those with clinical cancer cachexia (Selby *et al.*, 1987; Socher *et al.*, 1988). These results suggest that there may be other mediators of the cachexia in cancer patients.

We have utilised a murine colon adenocarcinoma (MAC16) to study the metabolic effects of the tumour on the host. At a body burden of only 2% this tumour produces a 33% reduction in host body weight, without a reduction in food or water intake (Bibby *et al.*, 1987). Cachexia in animals transplanted with the MAC16 tumours is associated with the presence of circulatory catabolic factors capable of breaking down both muscle and adipose tissue *in vitro* (Beck & Tisdale, 1987). Similar elevations in lipid mobilising activity have recently been found in the serum and urine of patients with clinical cancer cachexia (Groundwater *et al.*, 1990) and this study attempts to further characterise this activity.

Correspondence: M.J. Tisdale.
Received 1 October 1990; and in revised form 6 December 1990.

### Materials and methods

Pure strain NMRI mice were bred in our own colony. They were fed a rat and mouse breeding diet (Pilsbury, Birmingham, UK) and water *ad libitum*. Male mice (weight 26 to 28 g) were transplanted in the flank with fragments of the solid MAC16 tumour as previously described (Kitada *et al.*, 1981). Tumours were removed from animals with established weight loss and homogenised (10% w/v) at 4°C in Krebs-Ringer bicarbonate buffer, pH 7.6, and centrifuged for 10 min at 3,000 *g* to remove debris. Human colon carcinomas were obtained from the operating theatre of Dudley Road Hospital, Birmingham, UK (courtesy of Mr J. Neoptolemos) and were homogenised under the same conditions. The tumour supernatants were used for further characterisation.

### *Effect of starvation on lipolytic activity*

Male NMRI mice (10 to 12 weeks old) were starved for 24 h in metabolic cages. Water was provided *ad libitum*. Urine was collected for further analysis and body composition analysis was determined by the method of Lundholm *et al.* (1980) as previously described (Beck & Tisdale, 1987). Blood was removed from animals, using a heparinised syringe, by cardiac puncture, under anaesthesia with a mixture of halothane, oxygen and nitrous oxide. Glucose levels were determined on whole blood with the use of the o-toluidine reagent kit (Sigma Diagnostics, Poole, Dorset, UK). Plasma was prepared by centrifuging whole blood for 30 s and free fatty acid (FFA) levels were determined using a Walko NEFA C kit (Alpha Laboratories Ltd, Hampshire, UK). Urine creatinine concnetration was determined colourimetrically at 500 nm using a reagent kit (Sigma Diagnostics, Poole, Dorset, UK).

Six human control subjects volunteered to starve for 24 h, during which time only water intake was allowed. Urine and blood samples were provided, both prior to and after starvation, and were assayed for the presence of lipolytic activity.

*Exclusion chromatography*

Both mouse and human urine were fractionated using a Sephadex G50 column (1.6 × 30 cm) equilibrated with 10 mM phosphate, pH 8.0, and eluted at a flow rate of $15\,ml\,h^{-1}$. The void volume of the column (17 ml) was determined by blue dextran. The effluent from the column was collected in 1 ml fractions and the lipolytic activity of 0.5 ml samples was determined as described below. The column was calibrated with standards (cytochrome c, aprotinin, actinomycin D and rifampicin) of known molecular weight.

*Determination of lipid mobilising activity*

Mice (Strain BKW) were killed by cervical dislocation and their epididymal adipose tissue was removed and placed in isotonic saline, minced and incubated at 37°C for 2 h in Krebs Ringer bicarbonate buffer, pH 7.2, containing $2\,mg\,ml^{-1}$ of collagenase (Sigma Chemical Co., Dorset, UK) with prior gassing with 95% $O_2$: 5% $CO_2$. Digestion of the tissue was detected by the disappearance of intact pieces and an increased turbidity of the medium. Undigested material and non-adipose matter was removed by allowing the fat cells to float to the surface of the buffer and the infranatant was aspirated and replaced with fresh buffer. The washing procedure was repeated three times to remove all collagenase, non adipose cells and any endogenous hormones. After the final wash the cells were suspended in an appropriate amount of Krebs Ringer solution to give a density of $1.5 \times 10^5$ adipocytes $ml^{-1}$; the cell number being enumerated with a Neubauer haemocytometer.

Cell samples (1 ml) were removed, with continuous mixing to maintain a homogenous cell suspension, added to the appropriate test substance, gassed with 95% $O_2$: 5% $CO_2$ and incubated for 2 h at 37°C in a shaking water bath. Control samples containing adipocytes alone were also analysed to measure any spontaneous glycerol release. When assaying serum samples, a control (no adipocytes) was also included to measure the initial amount of glycerol present in the serum. Routinely samples of serum and urine (100 µl) were assayed in duplicate and the assay was repeated four to five times on each sample at different times. At the end of the incubatiion period, 0.5 ml of the incubation buffer was added to 0.5 ml of 10% (w/v) perchloric acid and the mixture was shaken to ensure deproteinisation. The precipitated protein was sedimented by centrifugation at 2,000 r.p.m. for 10 min, the supernatant removed and neutralised with 20% (w/v) KOH, after which the potassium perchlorate was sedimented by centrifugation (2,000 r.m.p., 10 min) and the volume of the supernatant was recorded and used to calculate the dilution factor. Assays on the supernatant were performed either immediately, or after storge at −20°C for between 18 and 72 h. The concentration of glycerol was determined enzymatically on 200 µl aliquots of the supernatant by the method of Wieland (1974). The results are expressed as µmoles glycerol released $ml^{-1}$ of serum or per mg creatinine in urine per $10^5$ adipocytes minus both the fat cell control value and the serum or urine control value.

*Statistical analysis*

All results are expressed as mean ± s.e.m. from at least three separate determinations. Differences were determined statistically using Student's *t*-test.

**Results**

Despite the fact that animals bearing the MAC16 tumour have a food and water intake not significantly different from non tumour-bearing controls, weight loss is observed, which increases as the tumour burden increases (Table I). We have attempted to compare the situation with starvation for 24 h where a similar weight loss is attained (Table I). The plasma levels of both glucose and FFA were reduced to a similar extent in both situations and body composition analysis showed a similar reduction in carcass fat, although the reduction in muscle dry weight, as measured by the thigh plus gastrocnemius muscles, was slightly less in the starved than in the cachetic animals.

The lipolytic activity of body fluids has been measured by the ability to liberate glycerol from freshly prepared murine epididymal adipocytes. Previous studies (Beck & Tisdale, 1987) have employed measurement of FFA release and substantially similar results are obtained with the two methods. The results in Table II show that both urine and plasma of

**Table I** Comparison of weight loss induced by starvation and by the MAC16 tumour on body composition and plasma glucose and FFA levels in male NMRI mice[a]

| Group | *Food intake (Kcal $day^{-1}$)* | *Weight loss (g)* | *Blood glucose (mg 100 $ml^{-1}$)* | *Plasma FFA (mg 100 $ml^{-1}$)* | *Body fat (g)* | *Muscle dry weight (mg)*[c] |
|---|---|---|---|---|---|---|
| Control | 14.9 ± 0.9 | – | 136 ± 5 | 29 ± 2 | 1.70 ± 0.09 | 90 ± 3 |
| Starvation[b] | – | 6.2 ± 0.2[e] | 106 ± 10[d] | 10 ± 1[e] | 0.54 ± 0.15[e] | 77 ± 3[d] |
| Cachectic[b] | 15.1 ± 0.6 | 5.4 ± 0.6[e] | 108 ± 11[d] | 10 ± 1[e] | 0.58 ± 0.11[e] | 70 ± 2[d] |

[a]The number of animals used in each group was six to eight. Initial weight 26 to 28 g. [b]The values refer to MAC16 animals 21 days after tumour transplantation while starvation was for 24 h. [c]Thigh plus gastrocnemius muscle dry weights. [d]$P < 0.05$ from control group. [e]$P < 0.005$ from control group.

**Table II** Activity of lipid mobilising factors in plasma and urine during starvation and cancer cachexia

| | *Urine lipolytic activity µmoles glycerol ($10^5$ adipocytes)$^{-1}$ (mg creatinine)$^{-1}$* | *Plasma lipolytic activity µmoles glycerol ($10^5$ adipocytes)$^{-1}$ (ml plasma$^{-1}$)* |
|---|---|---|
| A. *Mice*[a] | | |
| Control | 0.15 ± 0.065 | 0.020 ± 0.003 |
| Starvation | 0.14 ± 0.065 | 0.035 ± 0.005[d] |
| Cachexia | 1.06 ± 0.168[e] | 0.201 ± 0.030[e,f] |
| B. *Human* | | |
| Control | 0.056 ± 0.004 | 0.07 ± 0.02 |
| Starvation[b] | 0.048 ± 0.008 | 0.15 ± 0.03[d] |
| Cachexia[c] | 0.218 ± 0.023[e] | 0.37 ± 0.03[e,f] |

[a]The number of animals studied in each group was six to eight. [b]Starvation was for a 24 h period ($n = 4$). [c]This patient was a 53 year old female and an ovarian carcinoma and a weight loss of 31 kg ($n = 4$). [d]$P < 0.05$ from control group. [e]$P < 0.001$ from control group. [f]$P < 0.001$ from the starvation group.

cachectic animals bearing the MAC16 tumour display an enhanced lipolytic activity when compared with the values for non tumour-bearing controls. Although the total plasma lipolytic activity of animals after 24 h starvation was significantly higher than non-starved controls, the value was only one sixth of that found in animals bearing the MAC16 tumour. The lipolytic activity in the urine of starved animals was not significantly different from non-starved controls. This suggests that catabolism of body tissues in the cachectic state is associated with an enhanced lipolytic activity, which is not due to an elevation of hormones, normally associated with the catabolic state in starvation.

A similar situation was observed in a patient with clinical cancer cachexia, who also displayed an elevated plasma and urinary lipolytic activity compared with the normal controls (Table II). Again starvation significantly increased the plasma lipolytic activity of normal subjects, although the value was still significantly less than in the cachectic patient, but had not effect on the urinary lipolytic activity.

In order to investigate the molecular species responsible for the enhanced lipolytic activity of cachectic mouse urine an aliquot of urine from a mouse bearing the MAC16 tumour was subjected to exclusion chromatography using Sephadex G50. The lipolytic activity was mainly eluted at the void volume of the column, but was accompanied by three smaller peaks of activity with apparent molecular weights of 3, 1.5 and <0.7 kDa (Figure 1a). No comparable peaks of lipolytic activity were observed in the urine from a non tumour-bearing mouse (Figure 1c). The material eluting at the void volume of the column appeared to be an aggregate of the low molecular weight material, since treatment with 0.5 M NaCl and rechromatography on Sephadex G50 gave a broad range of activity peaks eluting between apparent molecular weights of 3 and 0.7 kDa, with no material eluting at the void volume (Figure 1b).

Similar lipolytic species with apparent molecular weights similar to that found in mouse urine were also observed in the urine of a cachectic cancer patient, when subjected to Sephadex G50 chromatography (Figure 2a). Urine from control human subjects displayed no corresponding peaks of lipolytic activity, even after starvation for 24 h (Figure 2b). These results suggest that the elevated lypolytic activity in the urine of animals bearing the MAC16 tumour and in patients with cancer cachexia is due to distinct molecular species.

Samples of fresh human colon adenocarcinomas, and in two cases the corresponding non-involved colonic tissue, were homogenised and assayed for lipid mobilising acitivity, without initially having the patient characteristics. Out of five samples assayed, only one (patient 2, Table III) was subsequently found to have lost weight, and the lipid mobilising activity of this tumour was twice that of the corresponding tumours from patients without weight loss. For those patients where the non-involved colonic tissue was also available, the lipolytic activity of the tumour extracts was significantly higher than normal tissue, even though no weight loss was observed.

### Discussion

Growth of the MAC16 tumour is associated with a progressive decrease in carcass lipids, although the animals consume normal amounts of food (Beck & Tisdale, 1987). Studies in rats have shown that the decrease in body fat cannot be accounted for by a decreased caloric intake, since pair-fed animals did not lose as much fat as tumour-bearing animals (Lundholm *et al.*, 1981). This suggests the involvement of a lipid mobilising factor in the catabolism of host adipose tissue in the cachectic state.

We have attempted to distinguish lipolysis in the cancer cachectic state from that occurring during starvation. Both states are associated with an increased plasma lipolytic activity, although the magnitude of the increase is higher in the cachectic state. Although a fat mobilising substance has been detected in the urine of man and some other mammals

**Figure 1** a, Sephadex G50 chromatography of a sample (1 ml) of urine from a mouse bearing the MAC16 adenocarcinoma with established weight loss. b, The fractions eluting at the void volume in A were concentrated, treated with 0.5 M NaCl and re-applied to the Sephadex column in A. c, Sephadex G50 chromatography of a sample (1 ml) of urine from a non-tumour bearing animal. Lipolytic activity is expressed as described in Methods. Ve = elution volume. Vo = volid volume.

during conditions of active fat catabolism (Kekwick & Pawan, 1963) we have been unable to detect an increased lipolytic activity in the urine of normal subjects (mice or humans) after acute starvation. Thus the enhanced lipolytic activity appears to be specific to the cancer cachectic state.

The ability of tumours to directly stimulate lipid mobilisation was first suggested by experiments with nonviable preparations of Krebs-2 carcinoma cells, which were able to induce fat loss in Swiss mice to a similar extent as the viable tumour cells (Costa & Holland, 1962). Later studies reported a lipid mobilising factor present in both the serum of mice bearing a thymic lymphoma and a patient with advanced cancer (Kitada *et al.*, 1981). The molecular weight of the material initially reported was 5 kDa, similar to that described in the present report, although later studies (Kitada *et al.*, 1982) suggested that activation only occurred on standing to give a high molecular weight material. Another lipid mobilising factor, Toxohormone L, has been isolated from the cell-free fluid of ascites sarcoma 180 and has been shown

**Figure 2** **a**, Sephadex G50 chromatography of a concentrated sample (100 ml) of urine from a male patient with teratoma and a total weight loss of 6.3 kg at the time urine was collected. **b**, Sephadex G50 chromatography of a sample (128 ml) of urine combined from control subjects after 24 h of starvation. Lipolytic activity is expressed as described in Methods.

to produce anorexia when injected into mice (Masuno *et al.*, 1981).

We have now shown that urine of both mice and humans with cancer cachexia appears to contain distinct molecular species with lipid mobilising activity which are absent during starvation. The true lipid mobilising fractions are probably of low molecular weight since the high molecular weight material is capable of being dissociated to the lower molecular weight forms with salt, suggesting an aggregation or non specific attachment to urinary proteins. We have recently shown (Groundwater *et al.*, 1990) that patients with cancer cachexia have increased levels of both serum and urinary lipid mobilising activity which increases with increasing weight loss up to a maximum body weight loss of 16–20%. Higher levels of lipolytic activity are also present in

**Table III** Lipid mobilising activity of human colon adenocarcinomas and non-involved colonic tissue

| *Patient* | *Sex* | *Age* | *Weight loss* | *Lipolytic activity*[a] |
|---|---|---|---|---|
| 1 | Female | 61 | 0 | 0.021 ± 0.004 |
| 2 | Male | 50 | 12.7 | 0.047 ± 0.006[c] |
| 3 | Male | 62 | 0 | 0.025 ± 0.007 |
| 4 | Female | 68 | 0 | 0.023 ± 0.006<br>(0.010 ± 0.002)[b] |
| 5 | Male | 68 | 0 | 0.021 ± 0.007<br>(0.002 ± 0.004)[b] |

[a]Results are expressed as μmoles glycerol released per ml tissue homogenate per $10^5$ adipocytes per h. [b]Values for non-involved colonic tissue. [c]$P < 0.001$ from patients 1, 3, 4 and 5 by Student's *t*-test.

a tumour extract from a patient with an adenocarcinoma of the colon with established weight loss, than from tumours from patients without weight loss, although preliminary data suggests that the lipolytic activity of the other tumours is higher than the normal colonic mucosa. Similar results were obtained in the MAC series of murine colon adenocarcinomas (Beck & Tisdale, 1987) where, although the cachexia-inducing MAC16 tumour had the highest lipolytic activity, significant activity was also found in the other tumour types. This suggests that tumours may have a system for obtaining lipids from the host, which may be utilised when *de novo* biosynthesis is insufficient to meet the metabolic demands.

Tumours obtain a substantial amount of preformed fatty acid from the host (Spector, 1975) either as circulating FFA (Mermier & Baker, 1974) or to a lesser extent from the triacylglycerols contained in plasma lipoproteins (Brenneman & Spector, 1974), despite the fact that tumours can synthesise fatty acids from glucose (Kannan *et al.*, 1980). Conditions which stimulate mobilisation of fatty acids from adipose tissue such as diabetes mellitus (Sauer & Dauchy, 1987*a*) and acute fasting (Sauer & Dauchy, 1987*b*) also stimulate the growth of the tumour. These findings suggest that the availability of nutrients derived from host fat stores may be rate limiting for tumour growth *in vivo*, which could provide some logic for the production by tumour cells of a substance capable, through some sort of receptor interaction, of mobilising lipids from host fat stores.

This work has been supported by a grant from the Cancer Research Campaign. S.A.B. gratefully acknowledges the receipt of a research studentship from the Cancer Research Campaign. We wish to thank Mr M. Wynter for the tumour transplantations.

### References

BECK, S.A. & TISDALE, M.J. (1987). Production of lipolytic and proteolytic factors by a murine tumour-producing cachexia in the host. *Cancer Res.*, **47**, 5919.

BEUTLER, B. & CERAMI, A. (1986). Cachectin and tumour necrosis factor as two sides of the same biological coin. *Nature*, **320**, 584.

BIBBY, M.A., DOUBLE, J.A., ALI, S.A., FEARON, K.C.H., BRENNAN, R.A. & TISDALE, M.J. (1987). Characterisation of a transplantable adenocarcinoma of the mouse colon producing cachexia in recipient animals. *J. Natl Cancer Inst.*, **78**, 539.

BRENNAN, M.F. (1977). Uncomplicated starvation versus cancer cachexia. *Cancer Res.*, **37**, 2359.

BRENNEMAN, D.E. & SPECTOR, A.A. (1974). Utilization of ascites plasma very low density lipoprotein triglycerides by Ehrlich cells. *J. Lipid Res.*, **15**, 309.

CHLEBOWSKI, R.T. (1985). Critical evaluation of the role of nutritional support with chemotherapy. *Cancer*, **55**, 268.

COSTA, G. & HOLLAND, J.F. (1966). Effects of Krebs-2 carcinoma on the lipid metabolism of male Swiss mice. *Cancer Res.*, **22**, 1681.

DE WYS, W.D., COSTA, G. & HENKIN, R. (1981). Clinical parameters related to anorexia. *Cancer Treat. Rep.*, **65**, 49.

GROUNDWATER, P., BECK, S.A., BARTON, C., ADAMSON, C., FERRIER, I.N. & TISDALE, M.J. (1990). Alterations of serum and urinary lipolytic activity with weight loss in cachectic cancer patients. *Br. J. Cancer*, **62**, 816.

INAGAKI, J., RODRIGUEZ, V. & BODNEY, G.P. (1974). Causes of death in cancer patients. *Cancer*, **33**, 568.

KANNAN, R., LYON, I. & BAKER, N. (1980). Dietary control of lipogenesis *in vivo* in host tissues and tumors of mice bearing Ehrlich ascites carcinoma. *Cancer Res.*, **40**, 4606.

KAWAKAMI, M. & CERAMI, A. (1981). Studies of endotoxin-induced decrease in lipoprotein lipase activity. *J. Exp. Med.*, **154**, 631.

KEKWICK, A. & PAWAN, G.L.S. (1963). Fat mobilizing substance. *Metabolism.*, **16**, 787.

KITADA, S., HAYS, E.F. & MEAD, J.F. (1981). Characterisation of a lipid mobilizing factor from tumors. *Prog. Lipid Res.*, **28**, 823.

KITADA, S., HAYS, E.F., MEAD, J.F. & ZABIN, I. (1982). Lipolysis induction in adipocytes by a protein from tumor cells. *J. Cell Biochem.*, **20**, 409.

LAHDEVIRTA, J., MAURY, C.P.J., TEPPO, A.-M. & REPO, H. (1988). Elevated levels of circulating cachectin/tumor necrosis factor in patients acquired immunodeficiency syndrome. *Am. J. Med.*, **85**, 289.

LUNDHOLM, K., EDSTRÖM, S., KARLBERG, I., EKMAN, L. & SCHERSTEN, T. (1980). Relationship of food intake, body composition and tumor growth to host metabolism in non-growing mice with sarcoma. *Cancer Res.*, **40**, 2515.

LUNDHOLM, K., EDSTRÖM, S., EKMAN, L., KARLBERG, I. & SCHERSTEN, T. (1981). Metabolism in peripheral tissues in cancer patients. *Cancer Treat. Rep.*, **65** (suppl. 5), 79.

MAHONY, S.M., BECK, S.A. & TISDALE, M.J. (1988). Comparison of weight loss induced by recombinant tumour necrosis factor with that produced by a cachexia-inducing tumour. *Br. J. Cancer*, **57**, 385.

MASUNO, H., YAMASAKI, N. & OKUDA, H. (1981). Purification and characterization of lipolytic factor (toxohormone-L) from cell-free fluid of ascites sarcoma 180. *Cancer Res.*, **41**, 284.

MERMIER, P. & BAKER, N. (1974). Flux of free fatty acids among host tissues, ascites fluid and Ehrlich ascites carcinoma cells. *J. Lipid Res.*, **15**, 339.

OLIFF. A., DEFO-JONES, D., BOYER, M. & 5 others (1987). Tumors secreting human TNF/cachectin induce cachexia in mice. *Cell*, **50**, 555.

SAUER, L.A. & DAUCHY, R.T. (1987*a*). Stimulation of tumor growth in adult rats *in vivo* during the onset of an acute fast. *Cancer Res.*, **47**, 1756.

SAUER, L.A. & DAUCHY, R.T. (1987*b*). Blood nutrient concentrations and tumor growth *in vivo* in rats: relationships during the onset of an acute fast. *Cancer Res.*, **47**, 1065.

SCUDERI, P., LAM, K.S., RYAN, K.J. & 6 others (1986). Raised serum levels of tumour necrosis factor in parasitic infections. *Lancet*, **ii**, 1364.

SELBY, P., HOBBS, S., VINER, C. & 7 others (1987). Tumour necrosis factor in man: clinical and biological observations. *Br. J. Cancer.*, **56**, 803.

SOCHER, S.M., MARTINEZ, D., CRAIG, J.B., KUHN, J.G. & OLIFF, A. (1988). Tumor necrosis factor not detectable in patients with clinical cancer cachexia. *J. Natl Cancer Inst.*, **80**, 595.

SPECTOR, A.A. (1975). Fatty acid metabolism in tumors. *Prog. Biochem. Pharmacol.*, **10**, 42.

WESDORP, R.I.C., KRUASE, R. & VON MEYENFELDT, M.F. (1983). Cancer cachexia and its nutritional implications. *Br. J. Surg.*, **70**, 352.

WIELAND, O. (1974). Glycerol UV Method, In *Methods of Enzymatic Analysis*, 3, Bergmeyer, H.U. (ed.) p. 1404, Academic Press: New York.